# BIOLOGICAL MARKERS OF AGING: CHRONIC STRESS AND COGNITIVE IMPAIRMENT IN RESPONDERS FROM THE WORLD TRADE CENTER

**DOI:** 10.1093/geroni/igz038.354

**Published:** 2019-11-08

**Authors:** Erica D Diminich, Sean Clouston, Stacey B Scott, Nikhil Palekar, Erica D santiago, Evelyn Bromet, Benjamin Luft

**Affiliations:** 1 Stony Brook University, New York, United States; 2 Stony Brook University, Stony Brook, New York, United States

## Abstract

Post-traumatic Stress Disorder (PTSD) is a stress related syndrome. Chronic PTSD has increasingly been associated with poor health outcomes, neurodegeneration and risk for cognitive impairment (CI). However, the biological mechanisms underlying the development and maintenance of symptoms and potential associations in accelerating aging are not well understood. The aim of this study was to evaluate whether specific biomarkers influence functional limitations and cognitive impairment in rescue and recovery workers (i.e. responders) from the attacks on the World Trade Center (WTC) in New York. Plasma biomarkers were collected during annual health and wellness visits at the WTC responder clinic between 2012 and 2014. Short Physical Performance Battery (SPPB) and clinical data were examined with prospective PTSD symptom scores collected during participant’s initial enrollment into the parent study as early as 2002. We examined the relationship between cardiovascular (Diastolic Blood Pressure, Systolic Blood Pressure, pulse rate), metabolic (Total Cholesterol, HDL cholesterol, Triglycerides, Glucose, Body Mass Index) and inflammation markers (Albumin, White Blood Count) with Post-traumatic Stress Disorder (PTSD), cognitive functioning (Montreal Cognitive Assessment) and frailty (Short Physical Performance Battery) in responders from the World Trade Center (WTC). We first examined correlations between biomarkers, PTSD symptom severity, PTSD dimensions, cognitive functioning and frailty. We then conducted multivariate regression analyses. In models adjusted for potential confounders, among N=1,045 responders, elevated PTSD was strongly associated with increased frailty, cardiovascular dysregulation and mild cognitive impairment. Current work is ongoing to identify trajectories of change in cognition with frailty and biological factors.

